# Fusing hyperspectral imaging and electronic nose data to predict moisture content in *Penaeus vannamei* during solar drying

**DOI:** 10.3389/fnut.2024.1220131

**Published:** 2024-01-24

**Authors:** Jiarong Wang, Wenxiu Wang, Wenya Xu, Huanjiong An, Qianyun Ma, Jianfeng Sun, Jie Wang

**Affiliations:** College of Food Science and Technology, Hebei Agricultural University, Baoding, China

**Keywords:** *Penaeus vannamei*, moisture content, electronic nose, hyperspectral imaging, data fusion, quality assessment

## Abstract

The control of moisture content (MC) is essential in the drying of shrimp, directly impacting its quality and shelf life. This study aimed to develop an accurate method for determining shrimp MC by integrating hyperspectral imaging (HSI) with electronic nose (E-nose) technology. We employed three different data fusion approaches: pixel-, feature-, and decision-fusion, to combine HSI and E nose data for the prediction of shrimp MC. We developed partial least squares regression (PLSR) models for each method and compared their performance in terms of prediction accuracy. The decision fusion approach outperformed the other methods, producing the highest determination coefficients for both calibration (0.9595) and validation sets (0.9448). Corresponding root-mean square errors were the lowest for the calibration set (0.0370) and validation set (0.0443), indicating high prediction precision. Additionally, this approach achieved a relative percent deviation of 3.94, the highest among the methods tested. The findings suggest that the decision fusion of HSI and E nose data through a PLSR model is an effective, accurate, and efficient method for evaluating shrimp MC. The demonstrated capability of this approach makes it a valuable tool for quality control and market monitoring of dried shrimp products.

## 1 Introduction

*Penaeus vannamei* shrimp, a leading species in global aquaculture with a production exceeding 6.5 million tons in 2021, is highly susceptible to spoilage due to physiological traits that foster microbial growth, such as high moisture content (MC) in tissues, minimal connective tissue, and active enzymes at room temperature (1). Its vulnerability to deterioration necessitates rapid processing and selling, commonly through freezing or drying. Drying is especially important in shrimp processing because the resultant low MC means that these aquatic products no longer depend on the cold chain and are easy to distribute (2). Among drying techniques, solar drying has proven to be a superior method that shortens processing time, enhances textural qualities, preserves nutrients, and reduces energy consumption, offering a green and efficient alternative to conventional natural and hot-air drying methods (3, 4).

Moisture is the main component of aquatic products. Real-time monitoring of MC and rapid prediction of quality changes are essential for maintaining the quality of *P. vannamei* during various stages of processing as improper drying can lead to diminished product quality through excessive shrinkage and hardening (5–7). While traditional oven-drying methods for measuring MC are reliable, they are also time-intensive and unsuitable for rapid analysis. Therefore, the *P. vannamei* drying industry requires a method that can monitor MC changes at different processing stages and quickly predict quality changes. Advancing technologies like Hyperspectral Imaging (HSI) significantly reduce the time needed for moisture analysis by concurrently capturing both imaging and spectral data, enabling the prediction of moisture content in meat products, such as in minced pork (8) and salted pork (9).

HSI is used to characterize the spectral information of shrimps, which is influenced by the stretching and vibration of chemical bonds. As moisture can influence the spectral characteristics of shrimp, the analysis of spectral information allows for the indirect determination of the shrimp’s water content. In addition. As an efficient tool, HSI adeptly captures color and texture variations in shrimps, a preliminary indicator of quality, yet its inability to discern odor—a critical quality determinant in seafood—highlights the necessity for a holistic evaluation method (10). Here, the combination of HSI and E-nose technologies becomes promising. Employing the electronic nose (E-nose) enables the detection of volatile compounds in shrimp, reflecting the influences of drying, enzymatic, and microbial processes alongside physicochemical interactions. The use of an E-nose allows for the detection of volatile compounds in shrimp, changes in which are influenced by enzymes and microorganisms, as well as by physico-chemical interactions. As moisture drives these activities, changes in them lead to changes in the concentration of specific volatiles. The sensors of E-nose can convert these detected chemical signals into electrical signals, allowing us to explore the relationship between these volatile compounds and MC. By employing multi-sensor data-fusion technology, data from HSI and E-nose can be effectively integrated, enabling a more comprehensive and accurate assessment of shrimp quality. This approach allows for the simultaneous acquisition of spectral information and volatile compound data of the shrimp, both of which are crucial factors in assessing shrimp quality (11). Furthermore, this also provides a new perspective for predicting the MC of shrimp.

Multi-sensor data fusion synthesizes diverse methods and tools to combine data from various sources. This innovative approach aims to harness the cumulative benefits of multiple data collection instruments, delivering enhanced accuracy data quality that supersedes the capabilities of a single technique. Embracing pixel-, feature-, and decision-level data-fusion strategies increases the reliability, robustness, and adaptive capacity of recognition systems. In pixel-level data fusion, comprehensive pre-processing is pivotal to seamlessly integrate multiple data sources. This foundational step ensures unblemished and harmonized data amalgamation. Feature-level fusion strategically selects specific variables from diverse datasets, amalgamated for enriched subsequent modeling and comprehensive analysis. In contrast, decision-level fusion involves the synthesis of various model results, culminating in a definitive and informed decision. This multifaceted approach to data fusion underscores its indispensable role in bolstering the precision and efficiency of modern recognition systems, solidifying their robust functionality across a spectrum of applications. However, no research to date has investigated the use of both HSI and E-nose for appraising the quality of solar-dried shrimp.

Therefore, the purpose of this study was to address this gap in the research using different data-fusion strategies. The specific objectives were to: (1) monitor changes in shrimp MC during solar drying; (2) acquire hyperspectral (color and texture) and E-nose sensor data, and then extract relevant variables (color, texture, and odor); (3) establish partial least squares regression (PLSR) quantitative MC prediction models with single-data or data-fusion (pixel-, feature-, and decision-level) strategies; and (4) compare model performance to determine the best fit and validate the HSI/E-nose combination technique. Our findings should benefit the aquaculture industry and contribute to an improved method for predicting MC when drying shrimp.

## 2 Materials and methods

**Figure 1:**
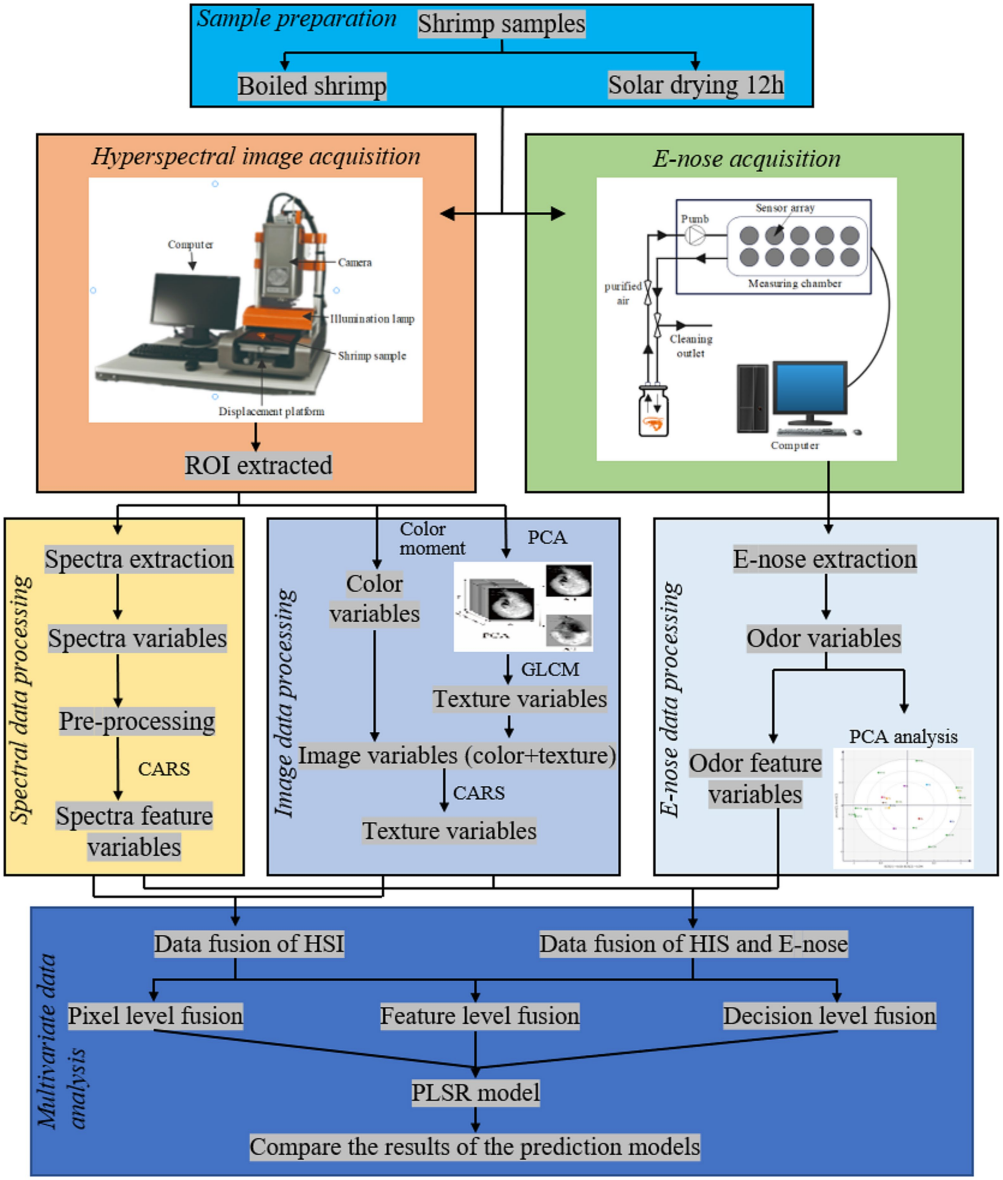
The primary experimental process's flowchart.

A summary of all experimental procedures is shown in the flowchart in Figure 1.

### 2.1 Sample preparation

Fresh shrimp (*P. vannamei*) were purchased at the Hebei Agricultural University Science and Technology Market in China. We selected fresh shrimp (average wet weight: 11.62 ± 2.2 g) with a complete body, uniform size, and disease-free status as the experimental material. The selected fresh shrimp were washed and boiled in salt solution 3% (w/v) at a 1:2 shrimp: salt solution ratio (w/w), boiled for 2 min, and then dried. The samples were spread on plastic mesh trays and dried using solar drying equipment (Supplementary Figure S1) for approximately 12 h. Shrimp were sampled at different stages, including boiled shrimp and after drying for 1, 2, 3, 4, 5, 6, 7, 8, 9, 10, 11, or 12 h. Eight samples were selected from each sampling site, resulting in 104 samples for subsequent HSI, E-nose, and MC analyses.

### 2.2 HSI acquisition and calibration

An HSI system, operating in reflectance mode, was employed to obtain hyperspectral images of shrimp samples. The system comprised an HSI workstation with a spectral range of 400–1,000 nm, a charge-coupled device camera (FX10, Specim Ltd., Helsinki, Finland) featuring a spatial resolution of 1,024 pixels and 224 spectral bands, two halogen lamps, and a computer equipped with HSI analysis software. Before collecting images, shrimp samples were placed on the electric replacement platform. Full-band images were captured at a resolution of 1,024 × 1,500 pixels and an imaging speed of 330 frames/s. Camera exposure time and conveyor belt speed were set to 50 ms and 7.5 mm/s, respectively. All shrimp samples across 13 groups were scanned, yielding 104 HSI encompassing both spectral (range: 400–1,000 nm, 224 bands) and spatial image data. The collected hyperspectral pictures were subjected to black-and-white correction to eliminate the effects of light-source nonuniformities and dark currents. To obtain a white calibration image, a standard white correction board was scanned under identical acquisition settings as the shrimp samples. The lens was covered to secure a black calibration image. The correction formula for HSI is given as Eq. 1.


(1) $$R=\frac{R_{\mathrm{raw}}-R_{\mathrm{black}}}{R_{\mathrm{white}}-R_{\mathrm{black}}}$$

where *R* is the reflectance image, *R*_raw_ is the raw HSI, *R*_black_ is the black calibration image, and *R*_white_ is the white calibration image.

### 2.3 E-nose data acquisition

The volatile compounds in shrimp samples were identified using a commercial portable E-nose system (PEN3, AIRSENSE, Germany), comprising a sampling unit, a sensor array, and pattern recognition software. The sensor array comprised 10 metal oxide sensors (Supplementary Table S1). The edible parts of shrimp at different drying stages were weighed and crushed with a blender. Then, 4.0 g of the sample was placed in a 15 mL headspace vial, with eight parallel sets prepared, and stored at 4°C. Samples were incubated at 30°C for 30 min, and then subjected to E-nose analysis. The parameters of the E-nose were set as follows: the sampling time was 100 s, flow rate was 400 mL/min, and cleaning time was 60 s with a 5 s zeroing time. Voltage data were recorded at between 90 and 91 s of stable state. These steps were repeated for each sample to acquire the E-nose data of 104 samples. The E-nose has 10 sensors; thus, each sample has 10 response values as odor variables for subsequent studies. The air chamber was ventilated and cleaned before each sample collection. To determine whether flavor characteristics could be used to distinguish between samples with different drying times, we employed principal component analysis (PCA) and hierarchical clustering analysis (HCA) to classify samples based on E-nose data.

### 2.4 MC measurements

After obtaining hyperspectral images and E-nose measurements, the MC per shrimp sample was analyzed using the oven-drying procedure specified in the National Standard of China (GB 5009.3-2016). Glass weighing bottles were dried to a constant weight in a 105°C oven. Samples were placed in the bottles and dried for 2 h at 103°C ± 2°C in an oven. After cooling to room temperature (25°C), each bottle was weighed and oven-dried for an additional hour. This process was repeated until the difference between the consecutive weighing results was less than 1.0 mg. The MC of shrimp samples was calculated using Eq. 2.


(2) $$X=\frac{m_{2}-m_{3}}{m_{2}-m_{1}}\times 100\%$$

where *X* (unit: g/100 g) is the MC of shrimp samples, *m*_1_ is the bottle weight, *m*_2_ is the weight of shrimp samples and bottles before drying, and m_3_ is the weight of shrimp samples and bottles after drying.

### 2.5 Data processing

#### 2.5.1 Extraction of raw spectral variables

For background correction, each region of interest (ROI) was extracted from the corresponding hyperspectral image in ENVI 5.2. A mask was constructed by subjecting each image to threshold segmentation at 685.5 nm (the wavelength that exhibited the largest reflectance difference between the shrimp sample and background). The binary masks of the shrimps were then used to extract ROIs for all spectral images at wavelengths between 400 and 1,000 nm. Subsequently, the average reflectance of all pixels within a given ROI was calculated and the spectrum of each sample was obtained.

#### 2.5.2 Extraction of raw image variables

Masks were applied to red, green, and blue (RGB) images synthesized from hyperspectral images captured at 647, 550, and 460 nm, respectively. Color and texture variables were extracted from mask images based on the color moment and gray-level co-occurrence matrix (GLCM) (12), respectively.

Color moments are a simple and effective way to represent the distributions of colors. Their advantages are that they have low-dimensional feature vectors and do not require quantization of the color space (13). Color distribution can be well-represented with low-order moments containing most of the relevant information (14), i.e., first-order (mean), second-order (standard deviation), and third-order (skewness). Consequently, RGB trichromatic values were transformed into hue, saturation, and value (HSV) trichromatic values. The first, second, and third moments of these six color components were employed to represent the color variables of distinct sample images, resulting in 18 color characteristics to represent between-sample differences. The GLCM-based analysis for extracting texture features is a typical statistical method with strong adaptability and robustness, primarily used for image detection and classification. First, masked images were subjected to PCA. Second, PC images with a cumulative variance contribution rate of up to 99% were selected to extract texture features (15), described using four basic values: contrast, correlation, energy, and homogeneity. We selected a *d* value of 1, meaning that the central pixel was directly compared with its adjacent pixels. Eigenvalues of the four directional matrices were calculated at 0°, 45°, 90°, and 135°, and then the average of the four eigenvalues was calculated as the final eigenvalue.

#### 2.5.3 Data pre-processing and feature variable extraction

Redundant data could potentially be present in the original hyperspectral imaging and E-nose datasets, arising from phenomena encountered during the experiment, such as light scattering, glossiness, and volatile aromatic compounds. Thus, both types of data require pre-processing. The sensor signals from the E-nose may be affected by baseline drift, and the pre-processing step helps eliminate this drift, normalize data, and remove superfluous and irrelevant information. This not only enhances the quality and stability of the data but also ensures the precision and efficiency of the model. Data were pre-processed using standard normal variate (SNV) and Savitzky–Golay derivative (S-G-Der) analysis. The former eliminates errors caused by different scattering levels between samples (16). The latter corrects for baseline drift and resolved peak overlaps in spectra (17).

Pre-processed data were multicollinear. To address this problem, redundant variables were eliminated by identifying a smaller set of optimal feature variables that most strongly affected prediction outcomes. These feature variables were extracted with competitive adaptive reweighting sampling (CARS). This method uses Monte Carlo sampling techniques to build several PLSR models and selects wavelengths with the fewest prediction errors as feature variables (18).

#### 2.5.4 Data fusion

Data fusion allows for the integration of data collected using various detection techniques. The strategy combines multiple data blocks into one model and improves predictive performance by working collaboratively between the individual blocks (19). Depending on the fusion structure, data fusion is typically divided into three levels: pixel-, feature-, and decision-level (20). The data fusion process of this study is shown in Supplementary Figure S2.

In pixel-level fusion, pre-processed data with the same number of lines were concatenated in series and then used as input variables for the samples. In feature-level fusion, feature variables were independently extracted from HSI and E-nose data and then concatenated into a matrix (21). This study selected feature variables (spectral, image, and E-nose characteristics) separately using CARS. In decision-level fusion, a separate PLSR model was established based on feature variables per source data type. Model outcomes were merged depending on decision criteria (typically mean, weighted mean, or majority vote) to yield the ensemble decision (22). Decision-level fusion was resolved with multiple linear regression, as follows:


(3) $$y_{ac}=b+k_{1}x_{\mathrm{spectra},pvc}+k_{2}x_{\mathrm{image},pvc}+k_{3}x_{E-\mathrm{nose},pvc}$$

(4) $$y_{\mathrm{dfpc}}=b+k_{1}x_{\mathrm{spectra},pvc}+k_{2}x_{\mathrm{image},pvc}+k_{3}x_{E-\mathrm{nose},pvc}$$

(5) $$y_{\mathrm{dfpv}}=b+k_{1}x_{\mathrm{spectra},pvv}+k_{2}x_{\mathrm{image},pvv}+k_{3}x_{E-\mathrm{nose},pvv}$$

In Eq. 3, *y*_ac_ is the actual value of the calibration set;

In Eq. 4, *y*_dfpc_ is the decision-fusion predicted value of the calibration set;

In Eqs 3, 4, *x*_spectra,pvc_ is the predicted values of the spectral calibration set; *x*_image,pvc_ is the predicted values of the image calibration set; *x*_E-nose,pvc_ is the predicted values of the E-nose calibration set;

In Eq. 5, *y*_dfpv_ is the decision-fusion predicted value of the validation set; *x*_spectra,pvv_ is the predicted value of the spectral validation set; *x*_image,pvv_ is the predicted value of the image validation set; and *x*_E-nose,pvv_ is the predicted value of the E-nose validation set;

In Eqs 3–5, *b* is the intercept of multiple linear regression; *k*_1_ is the weight of spectra; *k*_2_ is the weight of the image; and *k*_3_ is the weight of E-nose.

#### 2.5.5 Establishing and evaluating different models

The linear regression method PLSR is commonly applied in chemometrics (23, 24). This model fits the distribution of random variables to a linear equation by combining PCA with maximum correlation analysis and thus works best when variables are highly correlated (25). Using a 3:1 ratio, we selected 84 samples for the calibration set and 28 samples for the validation set. A regression model was established with PLSR to correlate spectral data, image data, and E-nose data with measured MC values. Data analysis and model evaluation were implemented in Matlab version 2014b.

In summary, regression model performance is comprehensively evaluated using various metrics including the root-mean-square errors of the calibration (RMSEC) and validation (RMSEV) sets, determination coefficients of the calibration ( $R_{c}^{2}$ ) and validation ( $R_{v}^{2}$ ) sets, and relative percent deviation (RPD) (26). The coefficient of determination (*R*^2^) reflects the correlation between the actual and predicted values, a closer approach to 1 indicates better regression performance. Additionally, a low root-mean-square errors (RMSE) signifies a high model accuracy by indicating a small error between actual and predicted values. High *R*^2^ and low RMSE values collectively point to an excellent model performance (27). Furthermore, the RPD, a ratio of sample standard deviation to its root-mean-square error, provides insight into prediction stability. A higher RPD value underscores better model stability, signaling a more reliable calibration model (28).

## 3 Results

### 3.1 Reference measurements of MC

**Figure 2:**
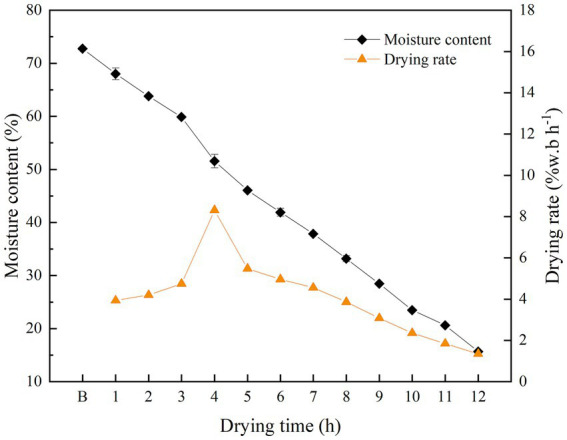
The MCs and drying rates of shrimp samples during the drying process.

The MC and drying rates of shrimp samples are shown in Figure 2. The MC showed clear and gradual decreases with extended drying times. The MC of boiled shrimp was 72.75% and decreased to 15.67% after 12 h of drying. The MC decreased slowly within the first 2 h of drying, with a drying rate of 4.2% w.b·h^−1^. The MC decreased faster from 2 to 7 h, with a maximum drying rate of 8.31% w.b·h^−1^ at 4 h. The drying rate decreased slowly after 7 h.

### 3.2 Spectral variable analysis

**Figure 3:**
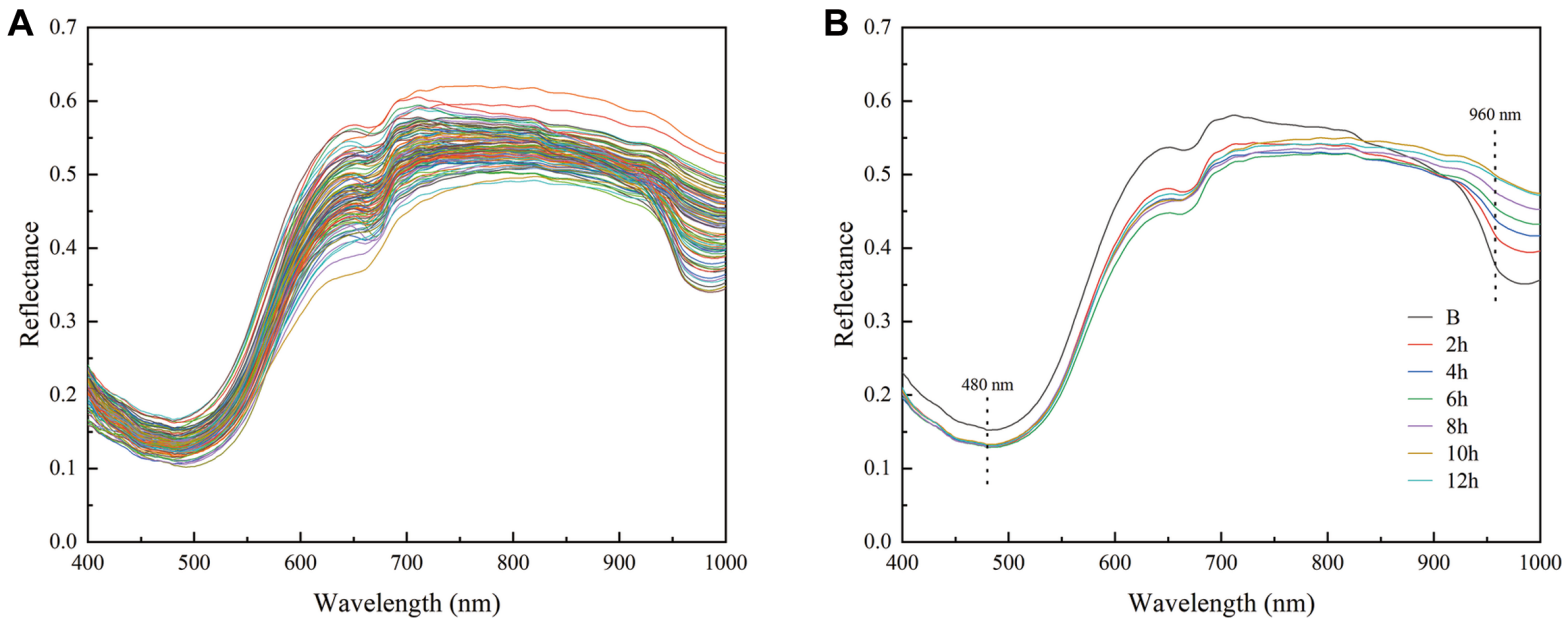
Reflectance spectra **(A)** average reflectance spectra of the ROIs for all samples and **(B)** reflectance spectra at different drying times (boiled, 2, 4, 6, 8, 10, and 12 h).

The average spectra of the ROIs in shrimp samples are shown in Figure 3. Figure 3A shows the original hyperspectral data over a wavelength range of 397.66 to 1003.81 nm with 224 spectral bands. The overall trends of the spectral curves of all shrimp samples were similar but the intensities of some bands were different (Figure 3A). These findings imply that the drying process leads to some significant changes in the shrimp samples that can be detected in the spectra. The spectral reflectance decreased slightly in the range of 400–480 nm, showed an upward trend beginning from 480 nm, and maintained a high level between 700 and 900 nm, after which it showed a small decreasing trend for different stages of shrimp samples between 900 and 1,000 nm. Figure 3B shows the representative reflectance spectra of shrimp at different drying times (2, 4, 6, 8, 10, and 12 h) and those of boiled shrimp; the reflectance of dried shrimp was lower than that of boiled shrimp between 400 and 830 nm, although a clear absorption peak appeared at 480 nm. In addition, the reflectance of the shrimp samples varied considerably at 960 nm, which was related to moisture and could have been caused by second overtone O-H stretches.

### 3.3 Image variable analysis

The color variables of shrimp sample images are shown in Table 1. With the first-order moments, all variables showed an increasing trend except for H; with the second-order moments, the R, S, and V variables showed increasing trends, and the G, B, and H variables showed decreasing trends; with the third-order moments, the V variable showed no change, the S variable showed an increasing trend, and the remaining variables showed insignificant trends. The observed alterations in the first-order moment variables may be attributed to the degradation of astaxanthin, which results in a darker and more intense coloration.

**Table 1:** Extracted image feature information of color.

Drying Times(h)	First order moments (mean)						Second order moments (standard deviation)						Third order moments (skewness)					
	R	G	B	H	S	V	R	G	B	H	S	V	R	G	B	H	S	V
Boiled	56.16	49.74	43.26	0.11	0.15	0.24	84.11	77.65	70.20	0.22	0.24	0.34	87.72	85.35	81.44	0.30	0.28	0.35
1	51.97	46.53	42.48	0.12	0.13	0.22	81.20	76.19	70.89	0.24	0.22	0.33	87.34	86.06	82.46	0.32	0.28	0.35
2	51.12	45.53	41.70	0.12	0.14	0.21	79.75	74.02	69.16	0.25	0.23	0.33	86.44	83.82	80.93	0.32	0.28	0.35
3	51.01	44.90	39.26	0.11	0.14	0.21	80.06	73.67	67.09	0.23	0.24	0.32	86.44	83.56	80.23	0.31	0.29	0.35
4	50.57	44.82	39.39	0.10	0.13	0.21	80.17	73.78	67.19	0.23	0.23	0.33	86.76	83.53	79.96	0.31	0.28	0.35
5	51.70	44.46	39.02	0.10	0.14	0.21	81.93	73.67	67.34	0.23	0.24	0.33	88.28	83.85	80.71	0.31	0.29	0.35
6	53.52	46.84	41.43	0.11	0.15	0.23	81.99	74.42	68.35	0.24	0.24	0.34	86.80	82.85	80.17	0.31	0.28	0.35
7	51.98	45.46	39.1	0.10	0.14	0.22	81.87	74.09	67.09	0.22	0.24	0.34	87.62	83.27	80.25	0.30	0.29	0.35
8	53.76	47.03	40.19	0.10	0.14	0.22	83.44	75.32	67.88	0.22	0.24	0.34	88.17	83.48	80.44	0.30	0.29	0.35
9	53.36	45.22	39.47	0.10	0.14	0.22	83.55	73.99	67.81	0.23	0.24	0.34	88.62	83.46	80.99	0.31	0.29	0.36
10	50.49	44.04	37.73	0.10	0.14	0.21	81.26	73.00	65.95	0.22	0.24	0.33	88.23	83.01	79.90	0.30	0.29	0.35
11	51.55	45.00	38.88	0.11	0.14	0.22	81.72	74.38	67.40	0.23	0.24	0.34	87.81	84.09	80.89	0.31	0.29	0.35
12	53.18	45.55	39.01	0.10	0.15	0.22	82.92	74.02	67.09	0.22	0.25	0.34	87.72	83.03	80.42	0.30	0.29	0.35

**Figure 4:**
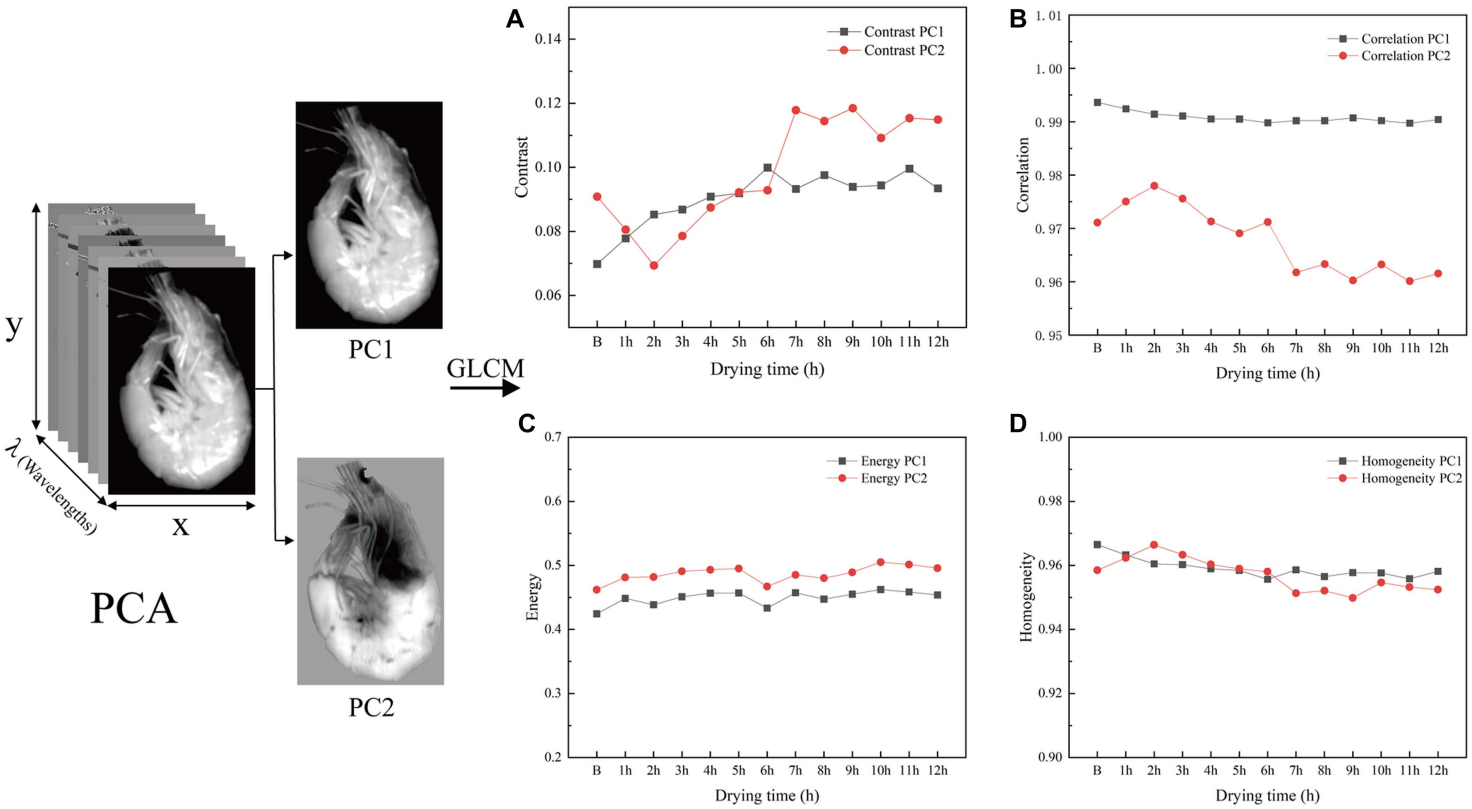
The PCA process and texture features of samples. **(A)**, **(B)**, **(C)**, and **(D)** stand for the change of contrast, correlation, energy, and homogeneity, respectively.

To extract texture variables, PCA was performed on the masked images of shrimp samples (Figure 4). The first principal component (PC1) explained >96% of the variance, and the first two principal components (PC1 and PC2) explained >99%. Therefore, texture variables (including contrast, correlation, energy, and homogeneity) were extracted with a GLCM for PC1 and PC2.

With increasing drying times, sample contrast tended to rise and then fluctuate smoothly, reflecting variation in muscle textural changes as the shrimp dried (Figure 4A). This trend likely stemmed from the gradual evaporation of water as shrimp meat dried, increasing muscle density and thus enhancing image contrast before it plateaued. The correlation coefficient for PC1 remained around 0.99, whereas that for PC2 fluctuated over a small range (0.96–0.98). In addition, PC1 explained far more of the variance than PC2, suggesting that the PC1 consistently represented the textural features of the shrimp throughout the drying process (Figure 4B). Energy fluctuations of PC1 (0.42–0.47) and PC2 (0.46–0.51) showed a small range and similar trends (Figure 4C) suggesting that the texture of the shrimp meat remained relatively uniform during the drying process. This may be because the drying process as a whole did not cause drastic changes in the texture of the shrimp meat. Variance was homogenous for PC1, reaching its minimum value after 6 h of drying; for PC2, variance ranged between 0.94 and 0.97 (Figure 4D).

### 3.4 E-nose variable analysis

**Figure 5:**
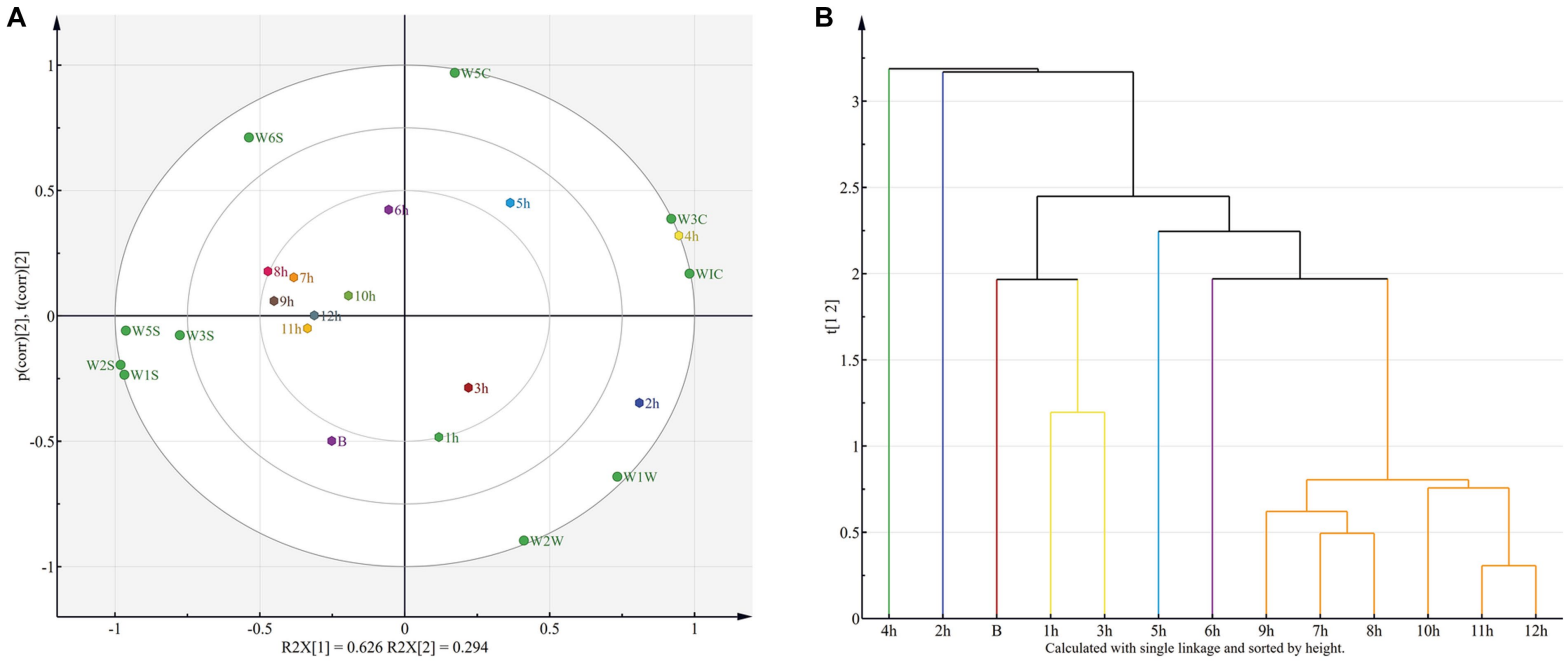
The PCA **(A)** and HCA **(B)** for all samples.

According to the results of PCA and hierarchical cluster analysis (HCA) of the electronic nose (E-nose) data (Figure 5), the cumulative variance contributions of the first principal component (PC1) and the second principal component (PC2) reached 92% (62.6 and 29.4%, respectively; Figure 5A). The samples were divided into seven distinct regions in the PCA analysis, which were identifiable in the component space. Specifically, Based on PC1, the 2 h samples were located at the rightmost end and in the first region; 1 and 3 h samples were in the second region; B samples were in the third region; 4 h samples were in the fourth region; 5 h samples were in the fifth region; 6 h samples were in the sixth region; and 7–12 h samples were in the seventh region. These regions indicate that different drying durations have a significant effect on the flavor of shrimp.

Based on HCA, seven principal groups were identified among the shrimp samples that corresponded to the seven regions revealed by PCA (Figure 5B). These results showed that the E-nose data of shrimp could be separated across the entire drying period and that drying duration influenced shrimp flavor characteristics. The results showed that the shrimp samples dried for 5–12 h had closer flavor characteristics than those dried for 2–4 h at the initial stage of drying (B, 1 h, and 3). This shows that this is a dynamic process, and some volatile compounds evaporated in the early stage of drying may lead to flavor differences between 2 h and 4 h. However, as the drying time exceeds 4 h, flavor rebalance may occur, which may make the flavor more consistent with the original flavor or the early stage of drying, as complex compounds break down into simpler compounds.

### 3.5 Performance analysis for the MC prediction model

#### 3.5.1 Prediction model based on raw variables

The results displayed in Table 2 indicate that the FD-SNV preprocessing technique significantly enhanced the predictive performance of the PLSR model based on spectral and image data. This model improved the RPD of spectral and image data by 57.32 and 12.22% compared with that of the PLSR model without pre-processed data. These results demonstrated the efficiency of FD-SNV as a pre-processing technique in reducing interference and improving prediction accuracy. The FD-SNV pre-processed spectral and image data were used for follow-up experiments. All pre-processed E-nose data were modeled and yielded RPD values >1.5 (Table 2). Among them, the PLSR model based on SNV pre-processing was the best predictor of MC, with $R_{c}^{2}$ , $R_{v}^{2}$ , and RPD of 0.7995, 0.7884, and 2.10, respectively. Therefore, SNV was chosen as the pre-processing method for E-nose data.

**Table 2:** The MC prediction models based on different pretreatments.

Models	Pre-processing	Variables number	LVs	Calibration set		Validation set		RPD
				$R_{c}^{2}$	RMSEC	$R_{v}^{2}$	RMSEV	
Spectra	RAW	224	9	0.9020	0.0577	0.8910	0.0700	2.18
	FD-SNV	224	10	0.9466	0.0425	0.9386	0.0479	3.43
	SD-SNV	224	7	0.9376	0.0460	0.9244	0.0528	3.08
	SNV	224	10	0.9349	0.0470	0.9336	0.0596	2.68
	SNV-FD	224	9	0.9339	0.0474	0.9336	0.0525	3.15
	SNV-SD	224	10	0.9470	0.0424	0.9367	0.0505	3.30
Image	RAW	26	10	0.6865	0.1031	0.6460	0.1108	1.47
	FD-SNV	26	9	0.6986	0.1011	0.6845	0.1101	1.65
	SD-SNV	26	9	0.6800	0.1041	0.6492	0.1119	1.49
	SNV	26	10	0.6764	0.1047	0.5838	0.1224	1.37
	SNV-FD	26	10	0.7333	0.0951	0.6666	0.1082	1.57
	SNV-SD	26	9	0.6669	0.1063	0.6400	0.1126	1.43
E-nose	RAW	10	6	0.7746	0.0874	0.7649	0.0961	2.02
	FD-SNV	10	6	0.7392	0.0940	0.6773	0.1096	1.64
	SD-SNV	10	7	0.7631	0.0896	0.6980	0.1043	1.71
	SNV	10	7	0.7995	0.0824	0.7884	0.0874	2.10
	SNV-FD	10	7	0.7916	0.0841	0.7763	0.0897	2.03
	SNV-SD	10	8	0.7674	0.0888	0.7345	0.0977	1.82

#### 3.5.2 Prediction model based on feature variables

**Figure 6:**
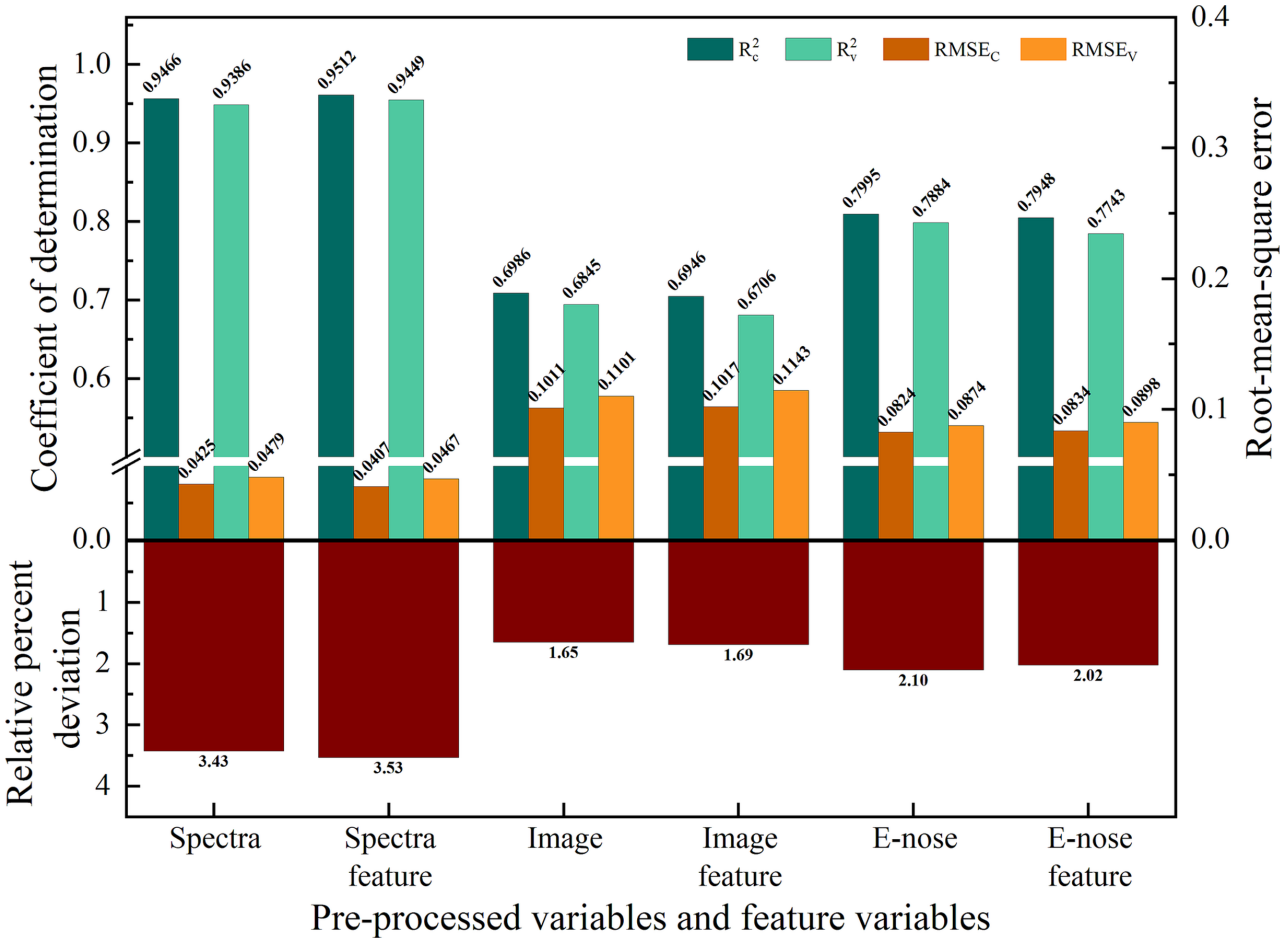
The MC prediction model based on a single information.

The extracted multivariate (high-dimensional) data contained many inter-band correlations, resulting in slow data processing, poor model accuracy, and weak model robustness (29, 30). The CARS method was used to select feature variables from spectral, image, and E-nose data, respectively yielding 14, 16, and 8 feature variables. Separate PLSR prediction models for shrimp MC were established based on these spectral, image, and E-nose feature variables (Figure 6). Fourteen bands (505.67, 542.91, 564.26, 588.36, 612.53, 737.25, 748.19, 786.58, 800.34, 850.06, 888.93, 891.71, 894.50, and 953.19 nm) were selected out of 224 bands in the spectrum. Compared with that of the PLSR model based on FD-SNV pre-processed spectra, the RPD of the PLSR model based on feature variables improved by 3.12%. For the PLSR model, basing the model on image feature variables improved the RPD by 2.27%. However, basing the model on E-nose feature variables did not improve predictive accuracy. Hence, only spectra and images are suitable for improving results when modeling with feature variables. The lack of improvement from E-nose feature variables may reflect a lack of features or low quality of features, resulting in the poor robustness of the model.

#### 3.5.3 Prediction model based on fusion variables

**Figure 7:**
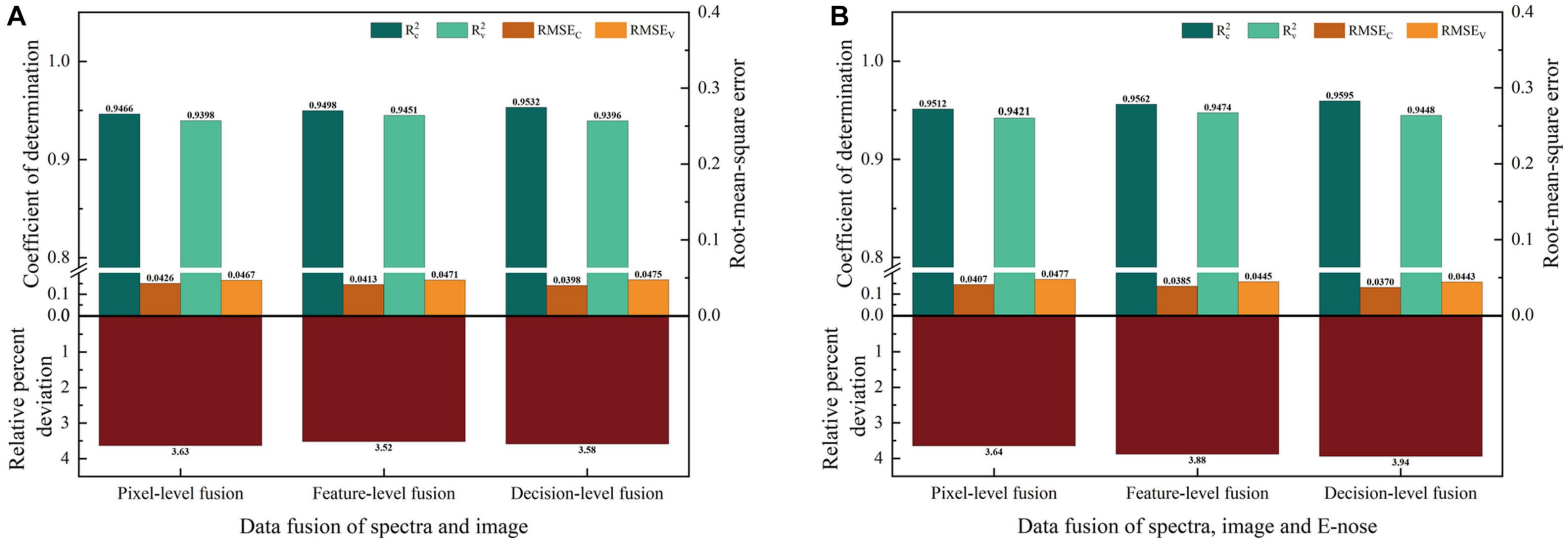
The MC prediction model based on data fusion. **(A)** Prediction results from spectral and image data fusion. **(B)** Prediction results from fusion of spectral, image, and E-nose data.

Figure 7A displays the results of MC prediction models based on data fusion of spectral variables and image variables (HSI). Pixel- and decision-level fusion models based on HSI outperformed models that contained only raw image variables or feature variables. However, pixel- and decision-level fusion models showed smaller improvements than the model based on spectral feature variables, with the RPD values only improving by 2.82 and 1.38% (Figures 6, 7A), respectively. The results were also slightly lower for the model with feature-level fusion than for the model based on spectral feature variables.

Figure 7B displays the results of MC prediction models based on data fusion of spectral, image, and E-nose variables (HSI and E-nose fusion). Prediction models based on three-level fusion of spectral, image, and E-nose data yielded better results than those based on a single variable or a single feature variable. Compared with the best model based on a single variable (a spectral feature), the RPD of models with pixel-level, feature-level, and decision-level fusion were improved by 3.09, 9.75, and 11.53% (Figures 6, 7B), respectively.

Predictions obtained by HSI and E-nose data fusion were compared with those obtained by the HSI technique alone (spectral and image data). HSI and E-nose fusion did not significantly improve model prediction under feature-level fusion, with the RPD increasing by only 0.26%. However, pixel- and decision-level fusion improved the model, increasing the RPD by 10.25 and 10.01%, respectively. Thus, the model with decision-level fusion based on spectral, image, and E-nose variables was the best predictor of MC. It exhibited the highest $R_{c}^{2}$ and $R_{v}^{2}$ values (0.9595 and 0.9448, respectively), lowest RMSEC and RMSEV values (0.0370 and 0.0443, respectively), and highest RPD value (3.94).

## 4 Discussion

This study introduced an innovative approach employing multi-sensor data-fusion technology to predict the MC in shrimp during solar drying, integrating data from HSI and E-nose sensors. The experiments demonstrate that the prediction accuracy of MC prediction model based on spectral data is the best. Concurrently, although the contribution of image data and E-nose data to MC prediction is relatively limited, its integration still fortifies the predictive capacity of the model. The results provided valuable insights into the potential and limitations of combining these technologies for enhanced MC prediction.

The HSI technique, which captured the spectral information, significantly contributed to the accuracy of MC prediction model. The changing spectral curves during the drying process, evidenced by the varying intensities at certain bands, provided crucial information correlating with the MC in the shrimp. For example, the third and second overtones of O-H stretching (absorbed at 750 and 980 nm, respectively) are linked to water content (31). This correlation underscores the critical role of spectral data in predicting MC, and the enhanced accuracy achieved with the fusion of spectral and image variables substantiates this assertion. Multiple previous studies have investigated the suitability of techniques we used here, either alone or in combination. For example, a previous study (32) utilized spectral data derived from near-infrared HSI to determine the chemical compositions of minced and whole pork, obtaining determination coefficients for MC prediction ( $R_{p}^{2}$ ) of 0.91 in the former and 0.58 in the latter. Another study (33) assessed the MC of frozen–thawed fish using spectral data generated from visible-near-infrared (Vis–NIR) HSI, obtaining an MLR model with $R_{p}^{2}$ = 0.9258 and root-mean-square error of prediction (RMSEP) = 1.12. Similarly, a study on scallops (34) developed an HSI method to ascertain MC at various dehydration stages, achieving optimal results with a wavelength-based PLSR model that yielded *R*_P_, RMSEP, and RPD of 0.9673, 3.5584%, and 3.7150, respectively. However, spectral data cannot accurately assess all changes that occur during drying. One possible reason for this limitation is that spectral data alone do not capture the complexity of interactions between different components within the food matrix as it dries.

Notably, E-nose data can effectively capture volatile compounds; however, in this case, it is less effective in improving MC predictions compared to spectroscopy. This may be owing to the insufficient correlation between the changes in volatile compounds during the drying process and MC, or these data may not provide valuable information for the prediction model. Despite the modest predictive performance of E-nose data when used independently, its fusion with HSI data, particularly the decision-level fusion, augments predictive accuracy. Future research might explore the potential of E-nose data in other aspects of shrimp quality assessment where the detection of volatile compounds plays a more central role.

Additionally, a significant improvement in the prediction model based on the fusion of HSI and E-nose variables emphasizes the value of integrating multiple types of data for enhanced prediction accuracy. A similar conclusion was reached by Ma et al. (6), who fused spectral data with image texture data to build an MC prediction model for pork that yielded $R_{v}^{2}$ = 0.9489 and RMSEV = 1.4736. In our study, the model accounting for odor variations yielded better results, which was consistent with the results of Cheng et al. (35), who fused spectral data, image data, and E-nose data to obtain a predictive model for MC in frozen–thawed pork with $R_{p}^{2}$ = 0.9533, and RMSEP = 0.3869. These results demonstrate that fusing multiple data types enhances prediction accuracy compared with using only one data type. Integrating diverse data sources enables the model to capture the complex relationships between different variables. Feature-level fusion can improve the predictive power of the model by reducing data redundancy but it may also reduce the stability of the model owing to an improper fusion strategy. Therefore, the model based on HSI feature-level fusion was not improved compared with the model based on spectral feature variables. Decision-level fusion enables independent model fitting without scaling adjustments (36). This strategy not only takes advantage of the complementary strengths of the information but also reduces the adverse effects of weak sensors by changing the result weights (35), which is also the main reason why the performance of the decision-level fusion model based on HSI and E-nose has been significantly improved in this study.

Understanding the importance of selected variables in decision fusion is crucial for developing effective predictive models. In this case, the weight assigned to each variable in the multiple linear regression equation provides insights into their significance. In the decision fusion based on HSI and E-nose, the multiple linear regression equation is y = 0.802×1 + 0.074×2 + 0.184×3−0.026. The equation reveals that the spectrum-based PLSR prediction model has the highest weight, followed by the weight of the electronic nose. The higher weight assigned to the spectrum-based PLSR prediction model suggests that certain characteristic spectra are strongly correlated with moisture changes. For instance, the paper mentions that the third and second overtones of O-H stretching are correlated with water content. This correlation explains why the spectrum-based PLSR prediction model holds greater importance in the decision fusion. On the other hand, the relatively lower proportion of the PLSR prediction model based on the electronic nose can be attributed to the instability of the test data collected by the electronic nose. Environmental factors such as air humidity can easily distort the data obtained from the electronic nose, leading to its lower weight in the decision fusion. This highlights the need for further refinement and stabilization of electronic nose data to enhance its effectiveness in moisture prediction. Furthermore, the small proportion of PLSR prediction models based on image data indicates that image data has limited significance in predicting moisture content. Although image data can capture changes in the appearance, shape, and color of shrimp, its correlation with moisture prediction is relatively low. Therefore, when analyzing different data types, it would be beneficial to explore and construct predictive models for other indicators to further study their changes and significance.

In conclusion, this study highlighted the effectiveness of employing a multi-sensor data-fusion approach for predicting the MC in shrimp during solar drying. However, the selection of suitable sensors and the discerning fusion of the corresponding data types remain paramount for optimal prediction performance. The contribution of E-nose data to predictive modeling is still limited. Further research can delve deeper into exploring other potential sensor technologies and data-fusion strategies for comprehensive and accurate evaluation of the quality of aquatic products from the drying process. Nuclear magnetic resonance and radio frequency sensing technology allow for non-contact, real-time monitoring of internal MC and distribution in food. This enables a better understanding and control over the drying process. In addition, this enhanced understanding is further complemented by employing advanced data analysis and machine learning algorithms, such as support vector machines and deep learning. These technologies not only facilitate the processing and analyzing of sensor data but also markedly augment the accuracy of predictions and assessments pertaining to the quality of dried food.

## 5 Conclusion

We successfully combined HSI and E-nose techniques to establish an accurate method for assessing shrimp MC. Our model is a unique tool for quality evaluation and market monitoring. During our creation of this MC prediction model, we compared multiple data-fusion techniques (pixel-, feature-, and decision-fusion). The results led us to conclude that models with a data-fusion strategy were superior to those based on a single variable. Additionally, decision-level fusion yielded better results than pixel- or feature-level fusion. Finally, incorporating E-nose data into the fusion model improved predictive accuracy. Our findings confirmed that the PLSR model based on decision fusion of HSI and E-nose data was the best predictor of MC. This model yielded the highest $R_{c}^{2}$ and $R_{v}^{2}$ , lowest RMSEC and RMSEV, and highest RPD. However, the model can be improved and its applications expanded. To this end, additional research should be conducted to simulate different drying temperatures and procedures to broaden sample variety. Furthermore, a tailored detection system with integrated portable HSI and E-nose sensors should be developed to support industrial applications.

## Data availability statement

The original contributions presented in the study are included in the article/Supplementary material, further inquiries can be directed to the corresponding authors.

## Author contributions

JiaW: conceptualization, investigation, and writing – original draft. WW: methodology and writing – review editing. WX: validation, formal analysis, and visualization. HA: software and data curation. QM: writing – review editing and supervision. JS: resources, conceptualization, and supervision. JieW: funding acquisition and project administration. All authors contributed to the article and approved the submitted version.
