# Supplementary material for: Fusing hyperspectral imaging and electronic nose data to predict moisture content in Penaeus vannamei during solar drying
Source: Front Nutr. 2024 Jan 24;11:1220131. doi: 10.3389/fnut.2024.1220131 (PMC10847239; doi:10.3389/fnut.2024.1220131)
Supplement: Supplementary file 1 [file Table_1.DOCX]

**Supplementary Table1** Sensors and their response characteristics.

| **Sensor** | **Response characteristics** |
| --- | --- |
| W1C | Aromatic compounds |
| W3C | Ammonia, aromatic compounds |
| W5C | Alkanes, aromatic compounds |
| W1S | Methane |
| W2S | Alcohols and some aromatic compounds |
| W3S | Alkanes |
| W5S | Nitrogen oxides |
| W6S | Hydrogen |
| W1W | Sulfides and terpenes |
| W2W | Organic sulfides and aromatic compounds |
