# Supplementary figures and images for: Fusing hyperspectral imaging and electronic nose data to predict moisture content in Penaeus vannamei during solar drying
Source: Front Nutr. 2024 Jan 24;11:1220131. doi: 10.3389/fnut.2024.1220131 (PMC10847239; doi:10.3389/fnut.2024.1220131)

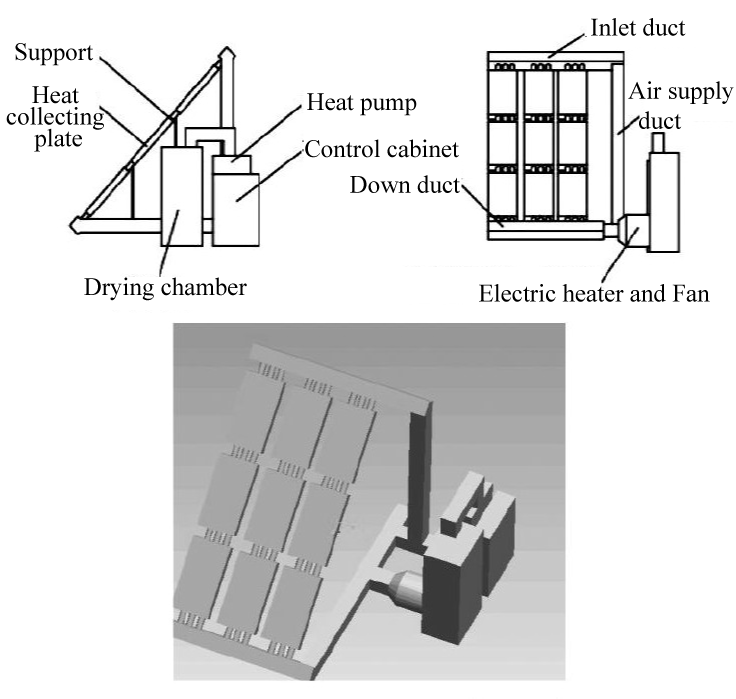

Supplement: Supplementary file 2 [file Image_1.TIF]
